# Impact of altered groundwater depth on soil microbial diversity, network complexity and multifunctionality

**DOI:** 10.3389/fmicb.2023.1214186

**Published:** 2023-08-03

**Authors:** Siteng Zhao, Xueyong Zhao, Yulin Li, Rui Zhang, Yanming Zhao, Hong Fang, Wenshuang Li

**Affiliations:** ^1^Northwest Institute of Eco-Environment and Resources, Chinese Academy of Sciences, Lanzhou, China; ^2^University of Chinese Academy of Sciences, Beijing, China; ^3^Naiman Desertification Research Station, Northwest Institute of Eco-Environment and Resources, Chinese Academy of Sciences, Tongliao, China; ^4^Tongliao Hydrology and Water Resources Sub-center, Tongliao, China

**Keywords:** semi-arid region, groundwater depth, soil multifunctionality, soil microbial community, relationship

## Abstract

Understanding the effects of groundwater depth on soil microbiota and multiple soil functions is essential for ecological restoration and the implementation of groundwater conservation. The current impact of increased groundwater levels induced by drought on soil microbiota and multifunctionality remains ambiguous, which impedes our understanding of the sustainability of water-scarce ecosystems that heavily rely on groundwater resources. This study investigated the impacts of altered groundwater depths on soil microbiota and multifunctionality in a semi-arid region. Three groundwater depth levels were studied, with different soil quality and soil moisture at each level. The deep groundwater treatment had negative impacts on diversity, network complexity of microbiota, and the relationships among microbial phylum unites. Increasing groundwater depth also changed composition of soil microbiota, reducing the relative abundance of dominant phyla including Proteobacteria and Ascomycota. Increasing groundwater depth led to changes in microbial community characteristics, which are strongly related to alterations in soil multifunctionality. Overall, our results suggest that groundwater depth had a strongly effect on soil microbiota and functionality.

## 1. Introduction

The sufficiency of water resources is crucial for arid and semi-arid regions, as the availability of water is closely linked to vegetation productivity and energy cycling (Karthe, 2018; Ma et al., 2022). Direct and indirect accessibility of water are considered as potential mechanisms explaining plant drought tolerance (Barron-Gafford et al., 2021; Ma et al., 2022). For example, The ability of *Prosopis tamarugo Phil.* to survive in the extremely arid ecosystem of the Atacama Desert is attributed to its ability to extend roots to access groundwater (Garrido et al., 2016). Alterations of soil water resources availability is the dominant driver factor of restoration, sustainability, and interactions of soil nutrients and vegetation community (Chai et al., 2019; Condon et al., 2021; Reed et al., 2022). Drought can lead to soil fertility and nutrient loss, which reduces the support for microbial habitats and soil ecosystem services, ultimately resulting in decreased productivity (Wall et al., 2004; Schlaepfer et al., 2017; Jia et al., 2020). The simultaneous occurrence of multiple ecological functions in an ecosystem should not be considered in isolation. Therefore, the use of integrated multifunctional indicators, such as soil multifunctionality that includes soil nutrients, soil water content, soil physical properties, soil temperature, soil pH, etc., will enhance our comprehension and prediction of the functions supplied by soils and habitats, as well as the responsiveness to biodiversity loss and ecological changes (Fanin et al., 2018; Garland et al., 2021).

Soil microorganisms play a vital role in driving nutrition utilization and biogeochemical cycling, supporting restoration of ecosystem (Bardgett and van der Putten, 2014; Delgado-Baquerizo et al., 2016; Crowther et al., 2019). Soil microbial diversity, composition, interactions, and functions are responsive to soil ecological alterations (de Vries et al., 2018; Guo et al., 2018). Desiccation induced by increasing groundwater depth has been shown to strongly modify the diversity of soil microbiota and functionality in different ecosystems (Hu et al., 2019; Zhang et al., 2020; Wu H. et al., 2021). Meanwhile, the soil microbial community is highly complex, comprising tens of thousands of species per gram of soil, and it should be noted that microbial diversity alone is insufficient to fully explain microbial functions (Van Der Heijen, 2008). Analyzing the relationships between individual microbial communities and their functional groups in co-occurring networks can reveal the interdependence arising from soil environmental heterogeneity (Banerjee et al., 2019). Network analysis can suggest whether microbial groups are more critical for maintaining network stability (Freilich et al., 2018; Zhang M. et al., 2023). The use of relevant metrics like betweenness and closeness centrality in assessing the complexity of microbial networks allows for the elucidation of the interconnectivity among operational taxonomic units (OTUs), which can be attributed to the heterogeneity of soil environmental conditions (Banerjee et al., 2019; Wagg et al., 2019; Martinez, 2023). This analysis provides insights into the influence of environmental factors on soil microorganisms and sheds light on the dynamics of microbial complexity, as well as how microbial interactions affect ecosystem function. Understanding the correlations between soil microbiota, soil multifunctionality, and drought (caused by groundwater depth increase) is crucial for the sustainability of ecosystems, as soil microbes are sensitive to soil quality.

Horqin Sandy Land, an ecologically fragile region, is one of the largest four deserts in northern China, with an area of approximately 139,300 km^2^, of which the desertification area is as high as 71,884 km^2^ (Wang, 2016; Luo et al., 2017; Zuo et al., 2020). The geomorphological features of the region are characterized by alternating sand dunes and slightly undulating lowlands (Luo et al., 2020). In recent years, expedited population growth and the resulting boosted requirement for food, housing, employment, and land have led to more ecological problems (Kooch et al., 2022), especially the decline of the ground water level (Maihemuti et al., 2021). As the temperature increases in the future, the potential enhancement in evapotranspiration will be more pronounced than that of precipitation, leading to more water shortage and exacerbating desertification or desertification in this region (Dai and Zhao, 2017; Su et al., 2018; Zhang Y. et al., 2023). The future changes in climate conditions may enhance the vulnerability of sandy ecosystems and strongly impact the biotic and abiotic processes in the soil environment. Groundwater depth may be a determinant regulating the changes in soil multifunctionality, soil microbial diversity and composition. Consequently, the objectives of our study were to systematically analyze the soil microbiota and soil properties in the agro-pasture crisscross region of the Horqin Sandy Land, considering variations in groundwater depth. We aimed to assess the responses of these soil factors to different groundwater depths and provide support for ecological revegetation and the implementation of groundwater conservation. Our research sought to explore the following objectives:

1. investigate the effect of altered groundwater depth on soil multifunctionality, soil microbial diversity, and composition;
2. analyze the co-occurrence networks and microbial functionality in different groundwater depth; and
3. evaluate the relative contribution of different groundwater depth on soil microbiota characteristics, soil moisture, and multiple soil functions and their coupling relationship among the four.

## 2. Materials and methods

### 2.1. Study area

The study area is in the Horqin Sandy Land of eastern Inner Mongolia, China (42°55′N, 120°42′E). The elevation in our study site is 327 m. This region belongs to a typical temperate continental monsoon climate, with an annual average temperature of 5.8–6.4°C and an average annual precipitation of 351.7 mm. The spatial and temporal distribution of rainfall is uneven, with approximately 80% of the precipitation occurring from June to September (Huang et al., 2022).

The region of study is characterized by low soil nitrogen content, with levels ranging from 0.057 to 0.199 g/kg. The bulk density of the topsoil layer (0–30 cm) ranges from 1.29 to 1.59 g/cm^3^ (Mao et al., 2012). The dominant vegetation species of this region are *Pennisetum centrasiaticum*, *Setaria viridis, Artemisia halodendron*, and *Caragana microphylla*. This study selected *Pennisetum centrasiaticum* and *Artemisia halodendron* because they are capable of surviving in various habitats, especially in areas with severe water shortage, and can help to maintain the stability of soil structure and prevent land degradation.

### 2.2. Experimental design

**Figure 1:**
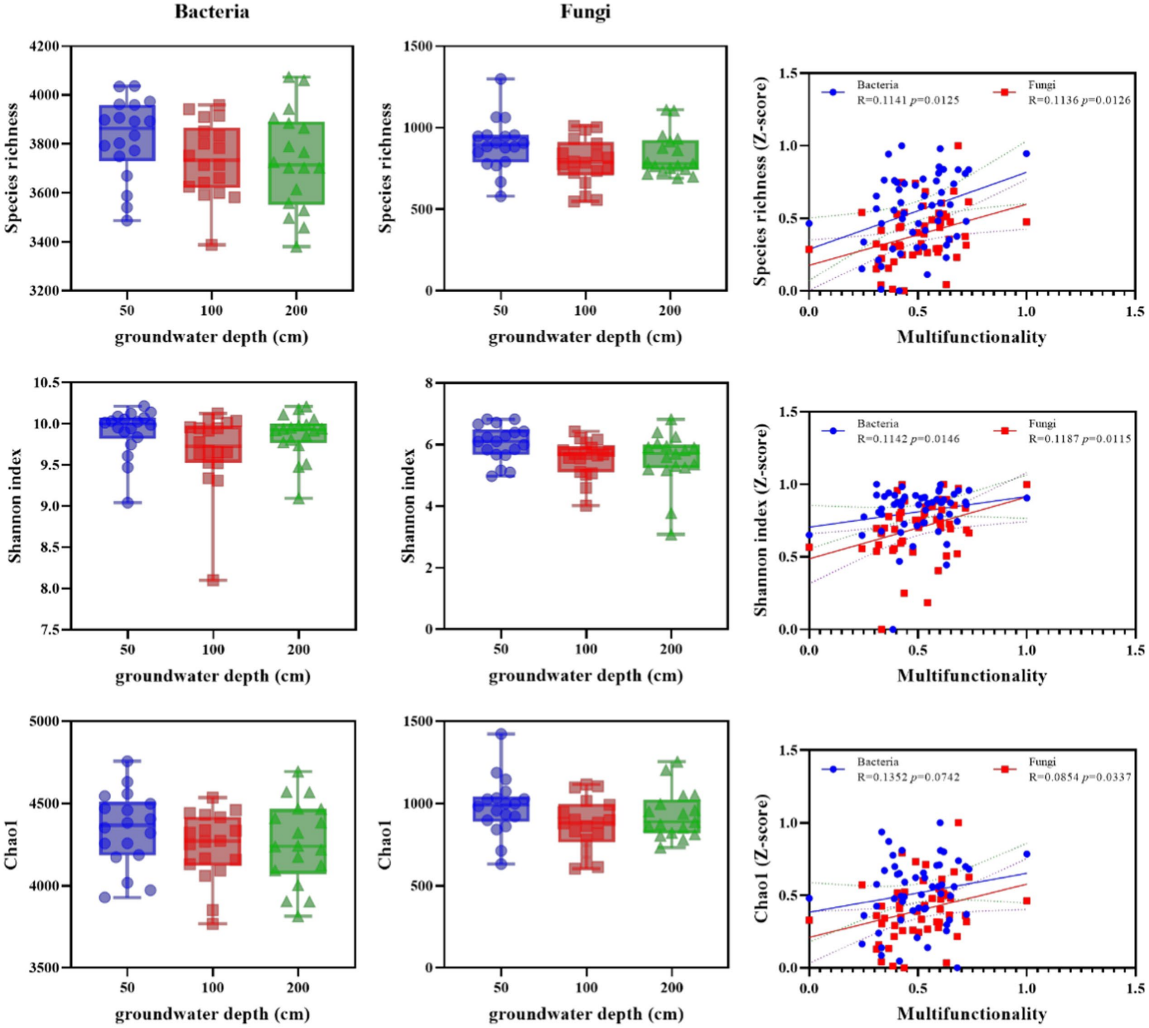
Alpha diversity of soil bacterial and fungal as altered by groundwater depth and the correlations of soil multifunctionality to alpha diversity. Diversity of bacterial and fungal communities (Observed species, Shannon index and chao1) in soils from 50, 100, and 200 cm groundwater depth plots, and the correlations of soil multifunctionality to diversity index. Box plots exhibit the first (25%) and third (75%) quartiles, and median, and the maximum and minimum observed values. Asterisks denote significant differences based on the Wilcoxon test. *p < 0.05, **p < 0.01, and ***p < 0.001.

This experiment began on June, 2017 at the Naiman Desertification Research Station (Su et al., 2021). As shown in Figure 1, three groundwater depth levels and two main species were used in the experimental manipulations (*Pennisetum flaccidum Griseb.* and *Artemisia halodendron Turcz.et Bess.*), with six replicates per each treatment. In 1 m^2^ concrete pools, an experimental operation was conducted, which included three treatment groups with groundwater depths of 50, 100, and 200 cm (Su et al., 2021). PVC shelters were set up to protect the cement pits from the rainfall. Establishment of a controlled experimental gradient was carried out to assess the effect of increasing groundwater depth on microbial diversity, network complexity, and multifunctionality of soil. Cement pits were buried to 1.0, 1.5, and 2.5 m according to each treatment gradient (Su et al., 2021). Stones with a diameter greater than 10 mm and a thickness of 10 cm were placed in cement pits, followed by sand to build the groundwater depth level. The bottom of each cement pit contained a 50 cm layer of stone–sand–water mixture. Adult individuals of *Pennisetum flaccidum Griseb.* and *Artemisia halodendron Turcz.et Bess.* were selected from the same sandy dune environment. The root system preserving the bud was excavated, and a root stem of equivalent diameter and length, possessing the bud, was selected. The selected rhizomes were then planted into the experimental pits.

### 2.3. Soil sampling and analyses

Sampling was conducted in August 2022. This study measured 11 soil variables, including soil moisture (SM), total nitrogen (TN), nitrate nitrogen (NN), ammonium nitrogen (AN), soil organic carbon (SOC), total phosphorus (TP), and pH in the 0–50 cm soil layer. The soil characteristics, such as nutrient availability, biogeochemical cycling, and microbial productivity, were assessed using these variables, and standard methods were employed for the measurements (Perez-Harguindeguy et al., 2013). Soil moisture (SM) was determined by oven-drying soil samples at 105°C to a constant weight. Soil organic carbon (SOC) and total nitrogen (TN) were quantified using the Walkley-Black and Kjeldahl method, respectively. total phosphorus (TP) was quantified through colorimetric analysis following digestion with sulfuric acid and perchloric acid, employing standard procedures. nitrate–nitrogen (NN) and ammonium–nitrogen (AN) were analyzed using a continuous flow analyzer (AutoAnalyzer-AA3, Seal Analytical, Norderstedt, Germany) following extraction with 2 mol/L KCl. Soil pH was measured in a soil:water extract (1:2.5) with a pH meter (Mettler Toledo, Germany). The C:N ratio was calculated as the mass ratio of SOC and TN, while the C:P ratio and N:P ratio were calculated as the mass ratios of SOC and TP, and TN and TP, respectively. Soil multifunctionality (MF) was evaluated based on seven soil variables (TN, NN, AN, SOC, TP, SM, and pH) according to the method described by Fanin et al. (2018). This method has been widely employed in various studies (Maestre et al., 2012; Wang et al., 2019). Before analysis, the normality of the data was tested using the Shapiro–Wilk test. Non-normally distributed data was transformed by taking the logarithm or square root to approximate normality. For variables with negative values, the variable was transformed to positive values by subtracting the minimum value of the entire dataset. Variables were then standardized to a scale of 0 to 1 and the mean of these transformed values was taken as the functional value for each pit.

### 2.4. DNA extraction, illumina sequencing, and data processing

Soil microbes’ DNA was extracted using FastDNA Spin Kits (Boaojingdian BiotECH Co., Beijing, China) and the primers 341F (5-CTAYGGGRBGCASCAG-3) and 806R (5-GGACTACNNGGGTATCTAAT-3) were used to amplify the V3-V4 regions of the 16S rRNA gene (Yu et al., 2005). The fungal community was targeted using the broad-spectrum primers ITS1 (5-CTTGGTCATTTAGAGGAAGTAA-3) and ITS2 (5-GCTGCGTTCTTCATCGATGC-3; Li et al., 2020a). The PCR reaction was performed using Phusion^®^ High-Fidelity PCR Master Mix (New England Biolabs) with a total volume of 15 μL, 0.2 μM of each forward and reverse primer, and approximately 10 ng of template DNA. The PCR cycling conditions included an initial denaturation at 98°C for 1 min, followed by 30 cycles of denaturation at 98°C for 10 s, annealing at 50°C for 30 s, extension at 72°C for 30 s, and a final extension step at 72°C for 5 min (Liang et al., 2020). The amplicons obtained from triplicate PCR reactions were combined for each sample and analyzed by electrophoresis on a 2% (*w*/*v*) agarose gel. The PCR products were purified using a Qiagen Gel Extraction Kit (Qiagen, Germany). Libraries for sequencing, with sample index tags, were generated using the Illumina TruSeq^®^ DNA PCR-Free Sample Preparation Kit (Illumina, United States). The quality of the library was evaluated using the Qubit^®^ 2.0 Fluorometer (Thermo Scientific) and the Agilent Bioanalyzer 2100 system. The sequencing was performed on the Illumina NovaSeq platform, generating 250-bp paired-end reads at the Boaojingdian Company in Beijing, China. The Tax4Fun2 package was used for potential functional annotation and metabolic prediction. A total of 5,534,451 reads were obtained from 66 samples, ranging from 64,032 to 95,768 reads per sample.

### 2.5. Statistical analyses

The alpha diversity indices, namely the Shannon index, observed species, and Chao1, were computed using the Mothur software 8 (Montagna et al., 2018). The beta diversity among different groundwater depths was calculated using Mothur software, analyzed with Bray–Curtis dissimilarity matrix and ANOSIM function. The *p*-values were modified using Bonferroni correction in *anosim* function. The sorting of Bray-Curtis distance analysis (PCoA) was performed using the capscale function implemented in the vegan R package (Delgado-Baquerizo et al., 2019; Li et al., 2020b). Soil samples were gathered from different groundwater depths for co-occurrence network analysis. In each of the four groups, taxonomic units that constituted more than half of the samples were used to calculate Spearman correlation coefficients. Benjamini and Hochberg FDR correction was applied to control for false discovery. Statistically strong associations were detected with Spearman’s *ρ* > 0.8 and FDR-adjusted *p* < 0.05 and included in the subsequent network construction (Benjamini and Hochberg, 1995). The topological parameters of the network, including the number of nodes, edges, betweenness centrality, and closeness centrality, were calculated using the igraph package (Lu et al., 2018). The impacts of soil properties on bacterial and fungal community composition and diversity at different groundwater depths were assessed using random forest and structural equation models (SEMs) in “randomForest” and “piecewise” packages, respectively (Li et al., 2020a; Kong et al., 2022). First, the relative contributions of groundwater depth, soil properties, and multifunctionality to bacterial and fungal species richness and community composition were measured using a random forest model, to identify the main response variables. Subsequently, a piecewise structural equation model was constructed to examine the direct and indirect impacts of groundwater depth, soil water content, and soil multifunctionality on the diversity and composition of bacteria and fungi. The Shipley’s d-separation test was conducted to check any missing pathways in the model, and a value of p greater than 0.05 indicated no missing pathways. Standardized coefficients for each pathway in each component model were reported, as well as the Fisher’s C statistic, AIC value, and BIC value for the entire model (Shipley, 2009; Kong et al., 2022).

All statistical analyses were performed using IBM SPSS Statistics (version 24) and R 4.0.5 (R Development Core Team, 2021).

## 3. Results

Significant decrease in groundwater level affected the physicochemical properties of soil and reduce soil multifunctionality (Table 1). Under deep groundwater conditions, the TN and SOC content, C:P, N:P, pH, and moisture in soil were all higher compared to shallow groundwater depths (*p* < 0.05). The content of NN increased initially and then declined as groundwater depth increased (*p* < 0.05). However, AN content was increased with the increasing groundwater depth (*p* < 0.05). Groundwater depth was negatively related to soil multifunctionality and soil multifunctionality was lower in deeper groundwater depth (Table 1).

**Table 1:** Effects of groundwater depth on soil properties.

Properties	Groundwater depth					*R* ^2^
	50 cm	100 cm	200 cm	*F*	*p*	
TN (g/kg)	0.3694 ± 0.0261a	0.3633 ± 0.0161a	0.2911 ± 0.0229b	3.867	0.0273	0.1317
NN (mg/kg)	131.6 ± 11.07b	145.8 ± 14.49a	97.24 ± 12.59c	4.979	0.0106	0.1634
AN (mg/kg)	15.68 ± 0.7663b	16.72 ± 1.0024b	21.47 ± 0.6641a	14.04	0.001	0.3551
SOC (g/kg)	3.619 ± 0.2573a	3.689 ± 0.2146a	2.585 ± 0.2175b	7.182	0.001	0.2198
TP (g/kg)	0.2039 ± 0.0054a	0.2022 ± 0.0062a	0.1967 ± 0.0074a	0.3579	0.7079	0.0134
C:N ratio	10.68 ± 1.016a	10.20 ± 0.4465a	10.02 ± 1.377a	0.1118	0.8944	0.0043
C:P ratio	17.78 ± 1.231a	18.84 ± 1.504a	13.24 ± 1.148b	5.218	0.0087	0.1699
N:P ratio	1.808 ± 0.1188a	1.843 ± 0.1065a	1.525 ± 0.1476b	1.843	0.1687	0.0674
SM %	4.808 ± 1.222a	2.120 ± 0.3607b	1.593 ± 0.1102c	5.449	0.0072	0.1761
pH	8.329 ± 0.0485a	8.108 ± 0.0637b	7.861 ± 0.0686c	14.81	0.001	0.3674
MF	0.6269 ± 0.0427a	0.5493 ± 0.0341b	0.3916 ± 0.0329c	10.59	0.001	0.2935

Different lowercase letters in the same row suggest significant differences at *p* < 0.05. TN, total nitrogen; NN, nitrate nitrogen; AN, ammonium nitrogen; SOC, soil organic carbon; TP, total phosphorus; SM, soil moisture; MF, multifunctionality of soils.

Soil microbial diversity was significantly altered by increasing groundwater depth in bacteria and fungi (Figure 1). The observed bacterial and fungal species richness, Shannon index, and Chao1 were significantly lower in deeper groundwater depth than in shallower depth. Nevertheless, the association among soil microbial diversity and MF was significant and positive (*p* < 0.05). Thus, losses of soil microbial diversity in altered groundwater depth were related with downward transitions in soil environmental functions.

**Figure 2:**
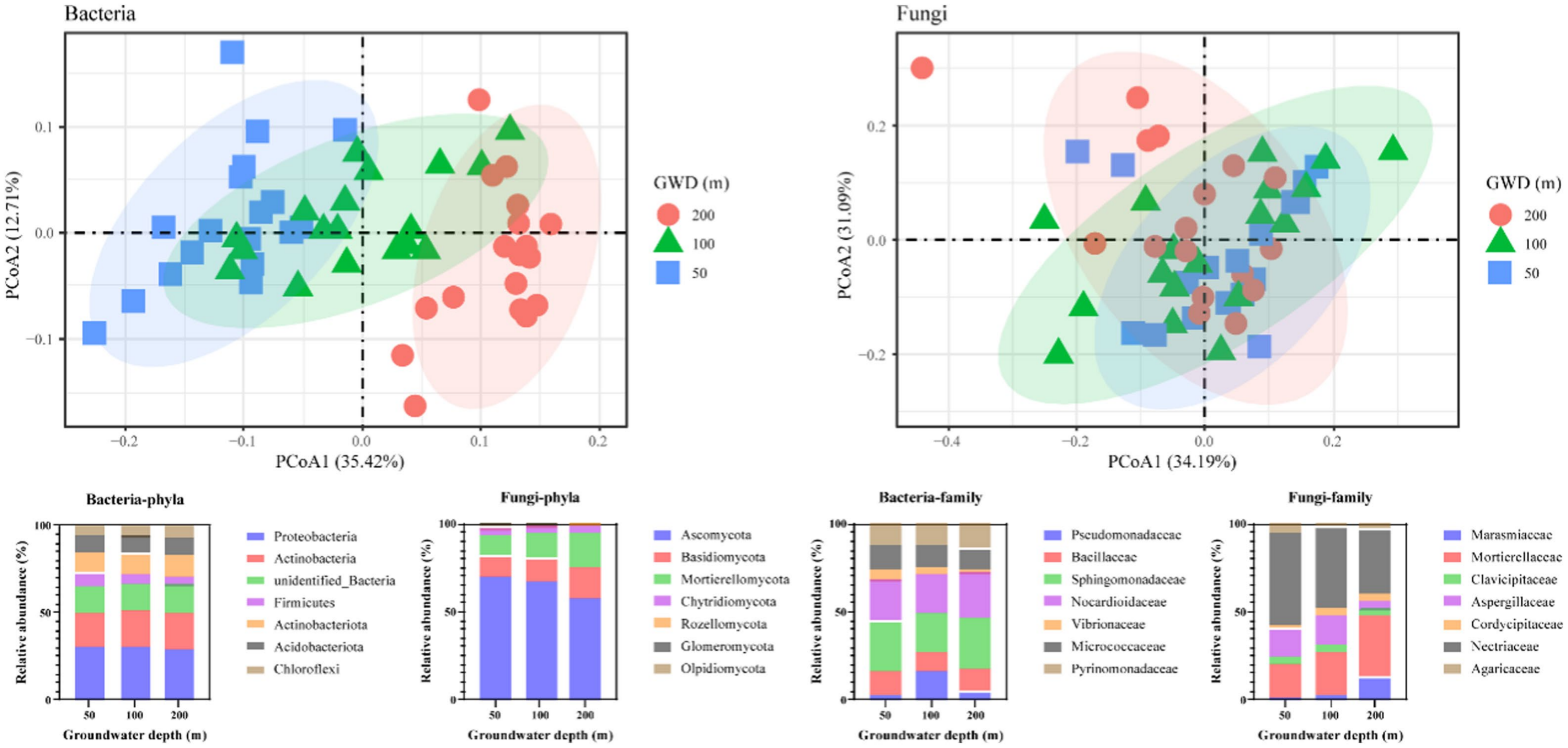
Soil microbial composition as influenced by groundwater depth on bacteria and fungi levels. Principal Coordinate Analysis (PCoA) plot of Bray–Curtis distances among groundwater depths (GWD = 50, 100, 200 cm); the relative abundance (%) of the phyla and family levels in soil dominant bacterial and fungal communities in three groundwater depth treatments.

Groundwater depth had a significant effect on the structure of soil microbial communities (Figure 2). Principle coordinate analysis (PCoA) suggested that soil microbiota strongly clustered following groundwater depth at bacteria level, which explained 12.71–35.42% of the total variation (Figure 2). Our results demonstrated that groundwater depth heavily affected the soil microbiota.

At the phyla level, the bacterial communities in the soil were primarily composed of Proteobacteria (29.86%), Actinobacteria (20.14%), Firmicutes (5.98%), Actinobacteriota (12.04%), Acidobacteriota (9.81%), and Chloroflexi (6.18%), while about 15.42–16.57% of the bacteria were unidentified; soil fungal communities were analyzed and found to be mainly composed of members of Ascomycota (65.18%), Basidiomycota (13.85%), Mortierellomycota (15.14%), Chytridiomycota (3.77%), Rozellomycota (0.71%), Glomeromycota (1.01%), and Olpidiomycota (0.19%), and these phyla accounted for more than 85% of the relative abundance of all identified phyla (Figure 2). With increasing groundwater depth, the relative abundance of Proteobacteria decreases in bacteria, while the relative abundance of Ascomycota decreases in fungi.

At the family level, Sphingomonadaceae (26.22%), Nocardioidaceae (23.71%), Bacillaceae (12.56%), Micrococcaceae (12.28%), and Pyrinomonadaceae (13.08%) emerged as a dominant family in the bacterial communities, constituting over 85% of the total bacterial sequences (Figure 2). The relative abundance of Sphingomonadaceae, Nocardioidaceae, and Bacillaceae was declined first then increased with the increasing groundwater depth. The dominant fungal family was Nectriaceae (44.71%), which was followed by Mortierellaceae (26.29%), Aspergillaceae (12.19%), Marasmiaceae (5.78%), Clavicipitaceae (3.96%), Agaricaceae (3.61%), and Cordycipitaceae (3.44%). The relative abundance of Nectriaceae and Aspergillaceae decreased strongly with growing groundwater depth, whereas the relative abundance of Mortierellaceae and Marasmiaceae boosted significantly with increasing groundwater depth.

**Figure 3:**
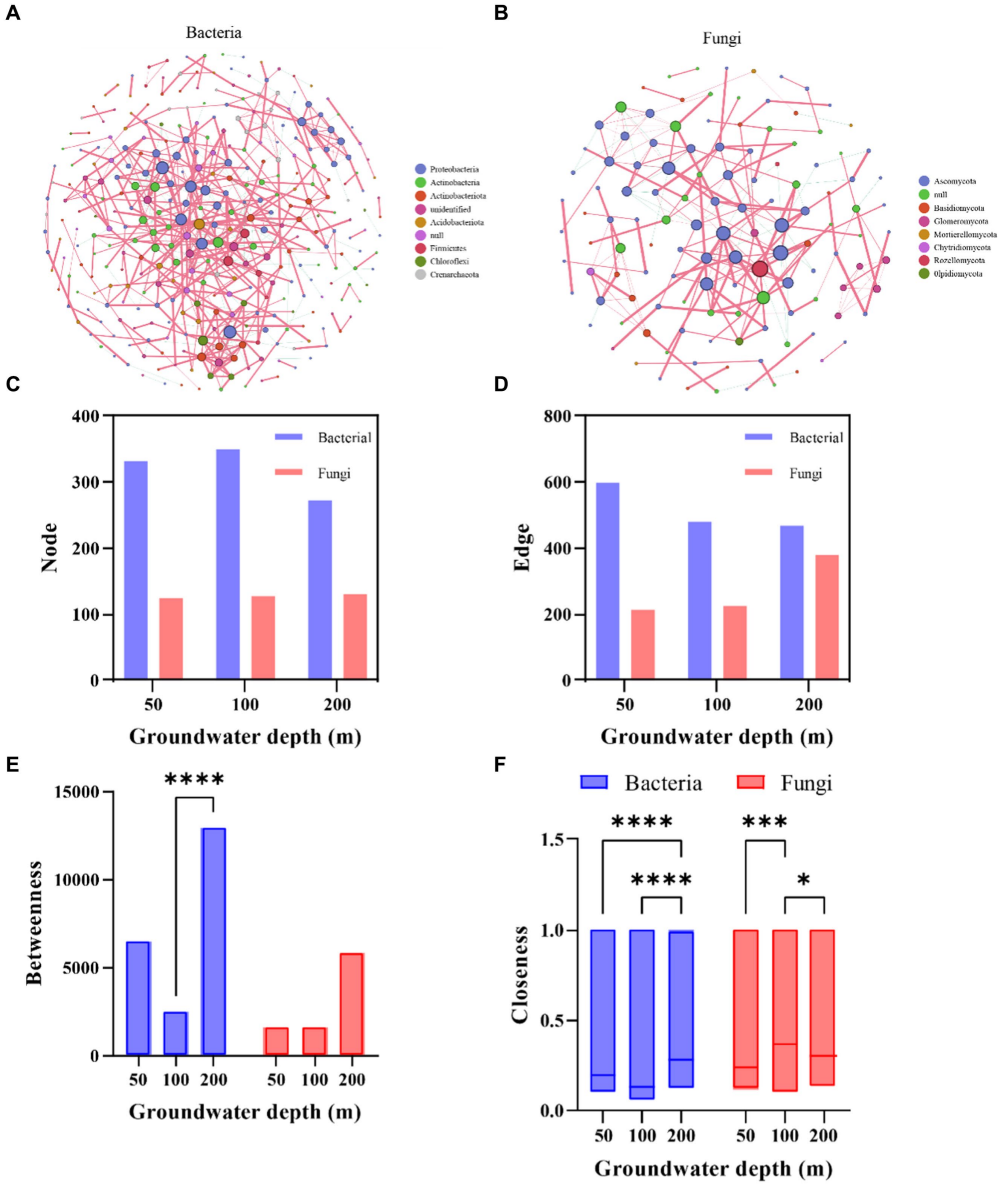
Co-occurrence networks for bacteria and fungi in different groundwater depth. Nodes in the network correspond to amplicon sequence variants (ASVs), and the color and size of each node indicate its phylum affiliation and the degree in the network. Red and green edges represent positive and negative interactions, respectively. Network properties for bacteria **(A)** and fungi **(B)**, comprised of node number **(C)**, edge number **(D)**, betweenness **(E)**, and closeness **(F)**. Asterisks denote significant differences based on the Wilcoxon test. ^*^p < 0.05, ^**^p < 0.01 and, ^***^p < 0.001.

The soil microbial community network exhibits apparent co-occurrence patterns (Figure 3). Groundwater depth had a negative influence on the complexity of soil bacterial co-occurring network, but increased the complexity of fungi (Figures 3C,D). For bacteria, the betweenness and closeness centrality were significantly greater in deeper depth than others, and the closeness centrality of fungi was significantly greater in 100 cm groundwater depth, indicating that the soil microbial network was less robust in the deeper groundwater depth than in the shallower (Figures 3E,F). These results suggested that increased groundwater depth impacted microbial correlations, and decreased the complication of soil bacterial community networks. Additionally, the effects of groundwater depth on soil microbial co-occurrence network were closely related to community composition and sensitive strains.

**Figure 4:**
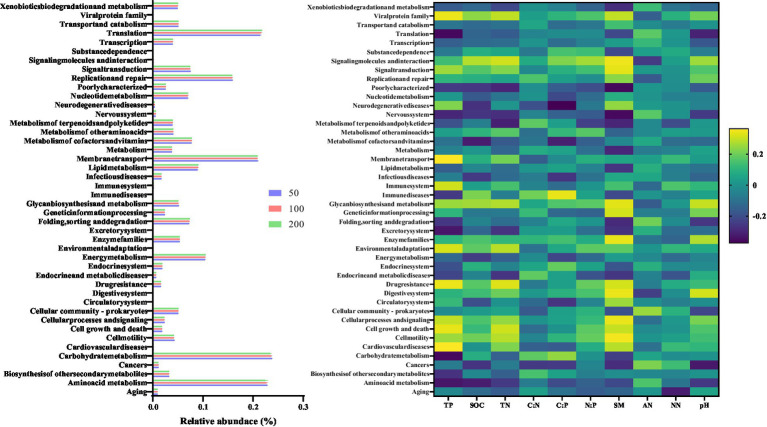
Variation in bacterial functions with altered groundwater depth. The relative abundance of the 44 bacterial functions predicted by Tax4Fun2-KEGG pathway (level 2). The Spearman’s correlation between predicted bacterial functions and soil properties.

Forty-two potential metabolic functional groups of the bacterial communities were predicted using Tax4Fun2 package based on KEGG pathway (Level 2) analysis (Figure 4). Increased groundwater depth decreased the values of most bacterial metabolic functional groups. For instance, the relative abundances of groups related to Amino acid metabolism, Biosynthesis of other secondary metabolism, Energy metabolism, Nucleotide metabolism, and Xenobiotics biodegradation and metabolism decreased after increased groundwater depth (*p* < 0.05). Meanwhile, most bacterial functional groups showed a strong negative relationship with soil properties.

**Figure 5:**
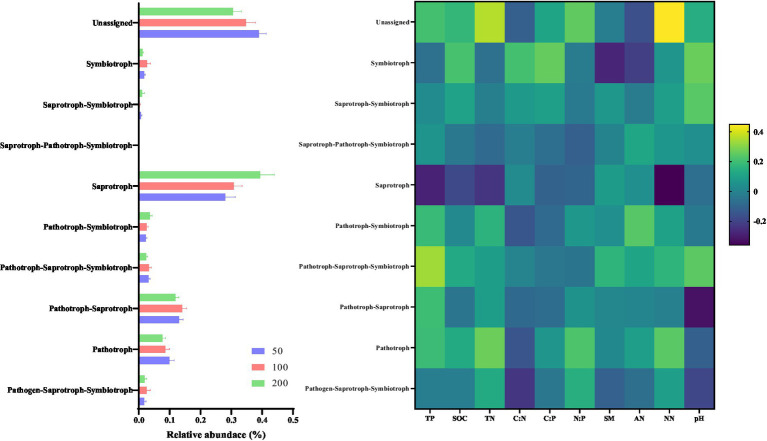
Variation in functions of fungal communities with altered groundwater depth. The relative abundance of the 10 functions predicted by FUNGuild. The Spearman’s correlation between predicted fungal functions and soil properties.

Derived from the results obtained from FUNGuild, the affected fungal functions are displayed in Figure 5. The relative abundance of fungal function which associated to Pathotroph decreased with increasing groundwater depth (*p* < 0.05). However, increased of groundwater depth improved the relative abundance of group associated to Saprotroph function (*p* < 0.05). Moreover, the relative abundance of fungal function related to Pathotroph was positively related to soil chemical properties (*p* < 0.05), while the relative abundance of Saprotroph group was negatively related to soil chemical properties (*p* < 0.05).

**Figure 6:**
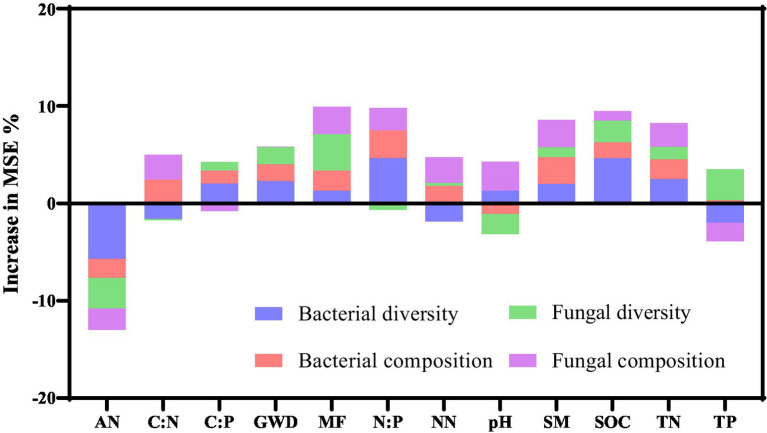
Primary predictors of the soil bacterial and fungal composition and diversity rely on random forest models. MSE, mean squared error; TN, total nitrogen; NN, nitrate nitrogen; AN, ammonium nitrogen; SOC, soil organic carbon; TP, total phosphorus; SM, soil moisture; MF, multifunctionality of soils.

Random forest analyses were employed to identify the key properties and multifunctionality of soil measures that were critical in predicting the soil microbial composition and diversity (Figure 6). In bacteria community, random forest models explained 25.47% of the variance in diversity and 12.46% in composition. The models based on random forest analyses were able to account for 14.75 and 10.75% of the variance in diversity and fungal composition, respectively. AN, N:P, and SOC were found to be the main predictors of bacterial diversity, while N:P, C:N, SM, MF, and TN were identified as the dominant predictors of bacterial composition. The main predictors of fungal diversity were found to be AN, MF, pH, SOC, and TP, while pH, NN, SM, and TN were the main predictors of fungal composition.

**Figure 7:**
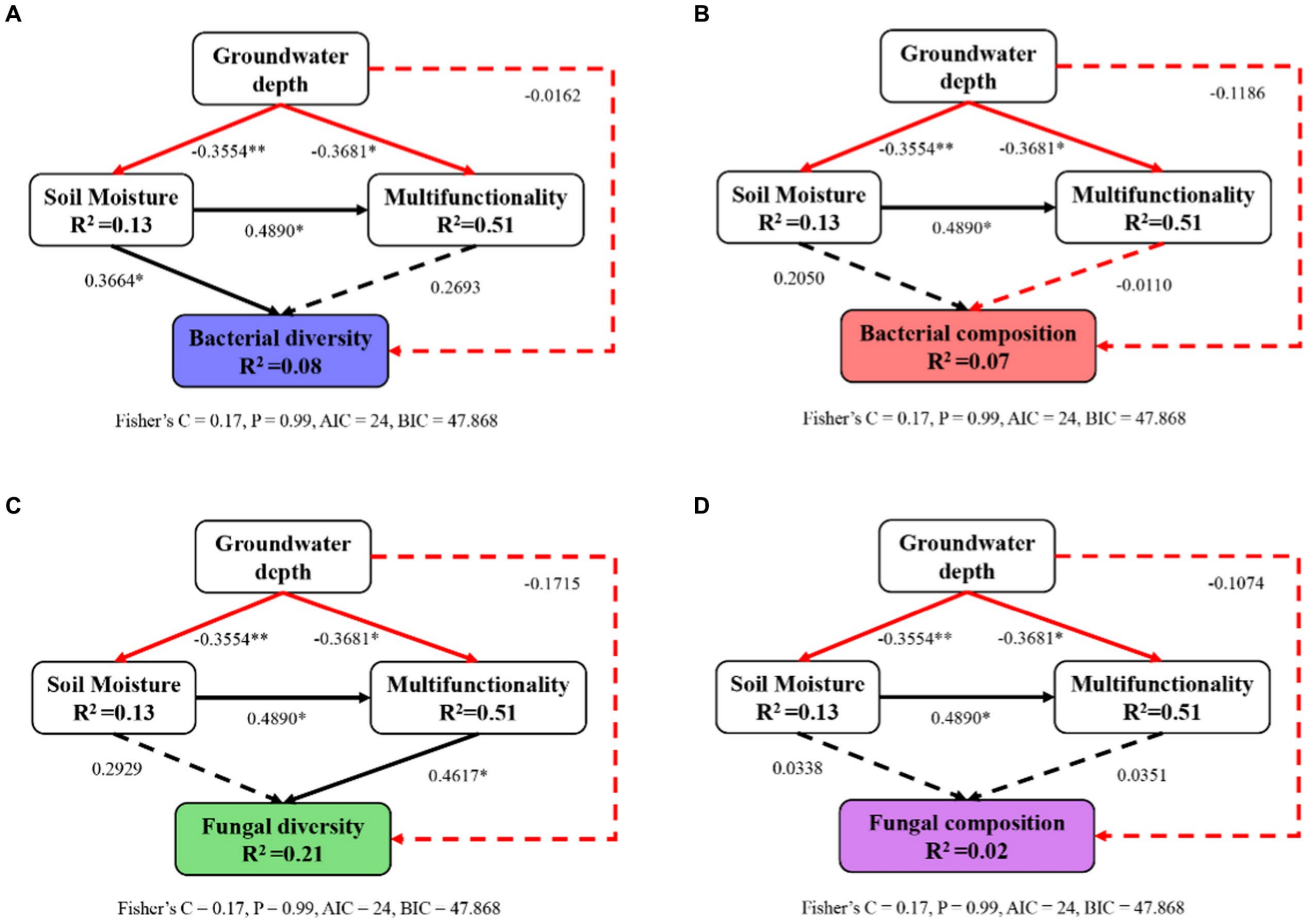
Impacts of groundwater depth, soil moisture, and soil multifunctionality on the diversity **(A,C)** and composition **(B,D)** of soil bacterial **(A,B)** and fungal **(C,D)** communities rely on a piecewise structural equation model (SEM). Solid lines indicate significant paths (^*^p < 0.05; ^**^p < 0.01); dashed lines indicate nonsignificant paths (*p* ≥ 0.05); black lines indicate positive effects; red lines indicate negative effects.

Structural equation modeling demonstrated that alterations in soil properties induced by changes in groundwater depth had a primary influence on the microbial diversity and composition (Figure 7). In contrast, the microbial composition was not significantly impacted by changes in soil moisture and multifunctionality (Figures 7B,D). Moreover, soil moisture and soil multifunctionality had significant and positive effects on microbial community diversity (Figures 7A,C). Increased of groundwater depth significantly reduced soil moisture and soil multifunctionality, whereas it had not direct effects on microbial community diversity and composition. Taken together, the increase in groundwater depth, which induces soil desiccation and degradation, can contribute to a decrease in microbial community diversity and composition in this semi-arid region.

## 4. Discussion

### 4.1. microbial diversity and soil multifunctionality

Our research on soil properties and microbial diversity with contrasting three groundwater depths revealed a decline in soil biodiversity and multifunctionality induced by the decreasing groundwater level. This finding has been extensively documented that the increase in groundwater depth can result in the reduction of soil nutrients, degradation of soil structure, reduction in soil moisture content, and decline in soil functionality, all of which have a detrimental impact on soil microbial diversity and composition (Prieto et al., 2012; Sun et al., 2020; Chen et al., 2021). Whereas, the impact of rising groundwater depth on soil microbiota has been relatively overlooked (Zhang et al., 2022), and it has not been clearly defined whether the alterations in soil microbial diversity caused by increasing groundwater depth are associated with a decline in multiple soil functions. The depletion in soil total nitrogen, soil organic carbon, and soil moisture by increasing groundwater depth could directly resulted in the loss of microbial diversity because reduced soil resource availability constrains the microbial metabolism and composition, furthermore decreased their supports on soil multifunctionality (Chen et al., 2020; Ye et al., 2023). Meanwhile, a previous study suggested that decreased soil organic matter content may improve soil thermal conductivity and a decline in soil heat capacity, then increase the daily variance of soil temperature (Abu-Hamdeh and Reeder, 2000; Balashov et al., 2023). Therefore, the indirect increase in soil thermal variability due to the deficiency of microbial diversity and multifunctionality is well-established, as many soil microbes are responsive to alterations in soil temperature (Karimi et al., 2018; Li J. et al., 2023).

### 4.2. Composition of soil bacteria and fungi

At the phyla level, the result showed that the relative abundance of Proteobacteria in bacteria decreased with increasing groundwater depth. The phylum Proteobacteria was found to be the most dominant bacterial group in the majority of the soil samples collected in our study. Zhang et, al (2016) found that the Proteobacteria is highly abundant and comprises fast-growing bacteria that can use a variety of resources, which are typically considered copiotrophic (Zhang et al., 2016). Meanwhile, the results indicated that the relative abundance of Actinobacteria increased with increasing groundwater depth. We also found increasing of groundwater depth significantly reduced soil moisture of 0–50 cm layers. The relative abundance of Actinobacteria was reported that decrease by the addition of water (Van Horn et al., 2014; Monteiro et al., 2023). In the fungal composition analysis, we observed a decline in the relative abundance of Ascomycota with increasing groundwater depth. The Ascomycota had previously been reported to have a strong positive correlation with the metabolism of organic substrates in rhizodeposition (Liu et al., 2020; Zhong et al., 2020). Consequently, the loss of available substrate and nutrients reduced the relative abundances of these microbial community phyla which was associated to increasing of groundwater depth. The relative abundance of Sphingomonadaceae increased with increasing groundwater depth at the family level. Our results also suggested that soil pH was negatively correlated with groundwater depth. A previous study demonstrated that Sphingomonadaceae was able to secrete dehydrogenase to balance soil pH (Gong et al., 2021). Increasing groundwater depth improve the relative abundance of Mortierellaceae in fungal community. The observed response pattern is likely attributed to the longer plant-water transport distance associated with deeper groundwater depth, which may prompt the plant to acquire available water resources by increasing the proportion of roots. The Mortierellaceae have previously been reported to have a positive relationship with root growth (Li et al., 2017). Overall, this study suggested that the alterations in soil microbial composition following an increase in groundwater depth were closely linked to the response of soil environmental conditions.

### 4.3. Microbial co-occurrence network

Our study also showed that the decline of groundwater level reduced the complexity of microbial co-occurring network. The observed decrease in soil microbial diversity may be attributed to the increasing groundwater depth leading to soil desiccation. Drought conditions are known to decrease microbial complexity as a result of their lower resistance to abiotic stress, and thus the loss of soil moisture caused by increasing groundwater depth could be a key driver in the observed decrease in soil microbial diversity (de Vries et al., 2018; Banerjee et al., 2019; Li Z. et al., 2023). Many studies have demonstrated that the complexity of soil microbial networks is positively associated with soil resources, such as soil moisture and fertility, and this interpretation aligns with the observed alterations in microbial composition and the response to soil conditions identified in our study (Guo et al., 2020; Hernandez et al., 2021; Kong et al., 2022; Zou et al., 2023). Besides, the robustness of microbial co-occurrence network was previously reported to be negatively characterized by betweenness and closeness (Figures 3C–F; Wu M.-H. et al., 2021). Our study showed that the responsive taxa of deeper groundwater depth had higher betweenness and closeness. This result suggested that these taxa may be responsible for driving the alterations in the microbial co-occurrence network (de Vries et al., 2018; Yang et al., 2023). Taken together, the results of our study provide additional evidence supporting the notion that deeper groundwater depth could have an indirect effect on the soil microbial co-occurrence network via soil desiccation.

### 4.4. Microbial functionality

For bacterial functionality, a decrease in the relative abundance of functional groups associated to amino acid metabolism and carbohydrate metabolism was showed in Figure 4. Many studies suggested that soil drought by increasing groundwater depth impaired these functional groups (Chen et al., 2012; Bromke, 2013; Kong et al., 2022). At a groundwater depth of 50 cm, the increase in soil moisture and soil nutrients could potentially mitigate the negative impact of drought on soil bacterial function. However, for deeper groundwater depth, drought caused by soil water stress may restrict microbial growth and hinder the availability of nutrients to bacteria by directly constraining bacterial dispersal within the pores of arid and sandy soil (Giannetta et al., 2018). We also found that the relative abundance of fungal Pathotroph decreased with increasing groundwater depth. The relative abundance of Pathotroph were suggested to be related with plant and root biomass, and deeper groundwater depth restrained growth of herb root (Levi et al., 2022). Although the relative abundance of Saprotroph was higher in the treatment with deeper groundwater depth, it is likely attributed to the increased soil heat capacity caused by drought. Consequently, the enhancement of fungal functionality, which is temperature-sensitive, may be affected (Qu et al., 2021; Li et al., 2022).

## 5. Conclusion

In summary, we explored the connections between groundwater depth, soil microbial diversity, microbial composition, soil moisture, and soil multifunctionality using the same soil and species at the same site in a semi-arid region. We found that soil moisture and multifunctionality changed by groundwater depth had different effects on soil microbial community (Figure 7). Careful consideration of such changes was necessary because in most arid and semi-arid climates, groundwater depth exceeds 50 cm (Chen et al., 2021; Trinidad Torres-Garcia et al., 2022). The results presented in our study suggest that deeper groundwater depth led to a depletion in soil moisture and soil properties, as well as a shift in microbial community structure and functionality. Essentially, soil microorganisms play a crucial role in supporting soil multifunctionality by promoting decomposition, nutrient cycling, and resource availability in different microenvironments (Delgado-Baquerizo et al., 2016, 2020). The present study highlights the significance of soil microbial communities in facilitating multifunctionality in arid environments, with diverse relationships between alterations in soil microbial diversity and composition and modifications in soil quality triggered by changes in groundwater depth. Additionally, as complex microbial communities are typically more resilient to environmental challenges than simpler ones, the observed reduction in microbial diversity and composition resulting from increased groundwater depth may have long-term undesirable impacts on soil functions (Fierer et al., 2003; Allison and Martiny, 2008; Crowther et al., 2015).

The importance of soil moisture and nutrient availability for the growth of drought-tolerant plants, especially in semi-arid and arid regions, where soil moisture plays a more significant role than rainfall, is widely recognized (McCulley et al., 2004; Li et al., 2021). Besides, the growing depth of groundwater is closely related to species mortality in response to drought (Goulden and Bales, 2019). Consequently, changes in water management may have a stronger effect on microbiota than changes in environmental factors. Furthermore, our results suggested that increasing groundwater depth which reduced soil moisture impaired soil microbial functionality. In semi-arid and arid regions, soil microbiota played a crucial role in regulating the availability of soil nutrients and water to plant roots. This study revealed that the reduction in dominant species in semi-arid and arid regions may be attributed not only to the depletion in soil water content but also to the impairment of multiple microbial functions resulting from drought (Peng et al., 2011; Fu et al., 2017; Stewart et al., 2017). Therefore, reduction of soil multifunction, soil water content, and soil microbial community caused by increasing groundwater depth together contribute to desertification. The investigation of the restoration of the soil microbiota and its functions in sandy soil under future groundwater conservation strategies is necessary.

## Data availability statement

The raw data has been successfully released with accession number PRJNA968046 and is available at: https://www.ncbi.nlm.nih.gov/bioproject/PRJNA968046.

## Author contributions

SZ: conceptualization, data curation, formal analysis, investigation, methodology, validation, visualization, writing—original draft, and writing—review and editing. XZ: funding acquisition, project administration, resources, supervision, validation, visualization, writing—original draft, and writing—review and editing. YL and RZ: conceptualization, formal analysis, investigation, methodology, resources, supervision, validation, visualization, writing—original draft, and writing—review and editing. All authors contributed to the article and approved the submitted version.

## Funding

This work was supported by the National Natural Science Foundation of China (No. 42177456), Transformation Program of Scientific and Technological Achievements of Inner Mongolia Autonomous Region (No. 2021CG0012), and the National Project on Science and Technology Basic Resources Survey of China (No. 2017FY100200).

## Conflict of interest

The authors declare that the research was conducted in the absence of any commercial or financial relationships that could be construed as a potential conflict of interest.

## Publisher’s note

All claims expressed in this article are solely those of the authors and do not necessarily represent those of their affiliated organizations, or those of the publisher, the editors and the reviewers. Any product that may be evaluated in this article, or claim that may be made by its manufacturer, is not guaranteed or endorsed by the publisher.
